# Supplementary material for: A systematic review of the association between perinatal depression and cognitive development in infancy in low and middle-income countries
Source: PLoS One. 2021 Jun 25;16(6):e0253790. doi: 10.1371/journal.pone.0253790 (PMC8232443; doi:10.1371/journal.pone.0253790)
Supplement: S1 File — (PDF) [file pone.0253790.s001.pdf]

Associations between perinatal depression and cognitive development in infancy in low- and middle-income countries

Matthew Bluett-Duncan, Thomas Kishore, Divya Patil, Helen Sharp

Citation

Matthew Bluett-Duncan, Thomas Kishore, Divya Patil, Helen Sharp. Associations between perinatal depression and cognitive development in infancy in low- and middle-income countries. PROSPERO 2018 CRD42018108589 Available from:
http://www.crd.york.ac.uk/PROSPERO/display_record.php?ID=CRD42018108589

Review question

Main Question: What is the evidence for an association between exposure to maternal perinatal depression and infant cognitive development in lower and middle-income countries?

Objective 1: Is maternal prenatal depression associated with infant cognitive development?

Objective 2: Is maternal postnatal depression associated with infant cognitive development?

Objective 3: Is there a difference in the strength of the associations found in prenatal or postnatal periods (obj:1 & 2)?

Objective 4: Is there evidence for independent or additive effects of prenatal and postnatal depression on infant cognitive development?

Objective 5: In the current literature, what other factors have been identified that may play a role in the transmission of risk from mother to infant?

Searches

Electronic search strategy:

The following electronic databases will be searched for relevant articles: PubMed, PsycINFO and CINAHL.

Databases will be searched from 1970 to August 2018. Studies should be published in English or provide sufficiently detailed English abstracts to enable comparison of the methods and main findings.

Keywords relating to the research questions have been identified. A combination of MeSH terms and free-text searches will be used, and search terms will be combined using Boolean operators. The search strategy for PubMed is available in the published protocol. The same search strategy will be applied to each electronic database for the sake of rigour but will be adapted for use with each database where necessary.

Grey Literature search strategy:

Grey literature will be searched in order to reduce the impact of publication bias. ProQuest will be searched using a combination of the following keywords: perinatal, prenatal, postnatal, antenatal, postpartum, depression, depressed, depressive, infant, infancy, child, children, cognitive, cognition, and LAMICs. Boolean operators will be used to combine terms.

Reference List search strategy:

Reference lists will be hand-searched for any relevant articles not recorded in electronic databases.

Types of study to be included

Inclusion:

The following observational studies will be included: prospective-cohort, cross-sectional, and case-control. Due to the emerging nature of research in this area in LAMICs, Randomised Control Trials will be included where they provide data regarding the control arm of the study or, failing that, they present baseline data at a cross-sectional level.

Studies should report a quantitative analysis of the association between perinatal depression and cognitive outcomes.

Exclusion:

Case-series, case-studies, RCTs without sufficient control arm or baseline data, studies only providing qualitative data, reviews.

Condition or domain being studied

The relationship between perinatal depression and infant cognitive development in low and middle-income countries (LAMICs).

There is a substantial body of global evidence supporting a significant association between perinatal disorders and a range of poor child outcomes. Both antenatal and postnatal depression have been found to be associated with significantly poorer infant cognitive outcomes (Stein *et al*., 2014)). While there have been a number of systematic reviews and meta-analyses that have found an overall effect of perinatal depression on cognitive outcomes in HICs (Liu *et al*., 2017, Kingston *et al*., 2012, 2015), and there are several reviews addressing this issue as part of a broader review on LAMICs, there are currently no systematic reviews that give a focused and in depth exploration of this association in LAMICs. This is an important issue to address as the prevalence of perinatal depression appears to be higher in LAMICs (Gelaye *et al*., 2016) and the poor economic conditions in these contexts can lead to higher risk of poor child outcomes (Parsons *et al*., 2012). In this review we aim to provide a comprehensive summary of existing research and knowledge in this area and present clear findings and suggestions for the direction of future research.

Participants/population

Inclusion:

- Women who at any point during pregnancy and up to one year postnatal were assessed for depression or depressive symptoms.

- Infants aged 0-36 months assessed for cognitive or language development.

- The only special interest group that will be included are mothers with perinatal depression or depressive symptoms. Otherwise, participants should be drawn from a community or general population sample.

Exclusion:

- Specific patient populations with any physical disease or disorder, either mother or infant, that will impact the cognitive development of the infant.

Intervention(s), exposure(s)

The exposure of interest is maternal perinatal depression or depressive symptoms.

“Perinatal depression is typically defined as a non-psychotic depressive episode of mild to major severity that occurs during pregnancy or up to 12 months postnatally” (Gelaye et al., 2016).

Inclusion:

Perinatal depression as diagnosed through a standardised diagnostic interview, or validated symptom

questionnaires or screening tools.

Exposure can be measured as a categorical or continuous variable. Where depression has been assessed at multiple time-points in the perinatal period, each will be reviewed in turn. Where both prenatal and postnatal depression have been assessed, both will be evaluated separately.

Exclusion:

Perinatal depression that is measured as part of a general mental health assessment where it is not possible to isolate the effect of perinatal depression from other symptoms or disorders on cognitive outcome.

Comparator(s)/control

Where appropriate data is available the comparator of interest will be infants whose mother's report no or low levels of depressive symptoms.

Context

Studies will be limited to those conducted in low- and middle-income countries according to the World Bank income classification. Studies that were low or middle-income at the point of publication will also be included.

Main outcome(s)

Infant cognitive development from 0-36 months of age. Studies that use quantitative methods to assess the impact of perinatal depression on cognitive performance of infants aged 0-36 months will be included in the review.

Cognitive development is defined as the development of cognition including, but not limited to, perception, concepts, memory, language, learning, problem solving, metacognition, and social cognition.

Cognitive development must be assessed using a direct (not implied), validated measure of cognitive ability. This includes general measures of cognitive development, measures of language development and any measures directly assessing any of the areas listed above. Language is included because it is closely related to, and heavily dependent on, cognitive development during infancy. Development may be assessed by an independent rater or a primary caregiver.

Additional outcome(s)

None.

Data extraction (selection and coding)

Title and Abstract Screening

Searches will be carried out by the lead author (MBD). Titles and abstracts will be screened by MBD and DP. Where it is not possible to screen studies based on the title and abstract, the study will be retained for full text screening. Any discrepancies will be discussed and re-examined until an agreement is reached. If no consensus can be found, a third reviewer will be consulted and will have the deciding vote. Reasons for exclusion will be recorded in reference management software.

Full Text Screening

Full text for studies that are deemed to meet inclusion criteria will be retrieved electronically where available. Resources not available will be pursued through institutional sharing agreements. Full text articles will then be checked against specified inclusion and exclusion criteria. Reason for exclusion will be recorded in reference management software. A flow-chart showing the study selection process for both stages will be provided.

Data Extraction

To organise and facilitate data comparison, tables will be created by extracting data from each study into an SPSS database. Data will be extracted independently by MBD and DP. Any disagreements will be discussed, and if agreement is not found, a third reviewer will be consulted. If relevant information is not

provided in the study article, the authors of the article will be contacted for the required information. The data extraction form will be piloted to test its efficiency and accuracy.

Data to be extracted will include the following: country, study design, aims/hypotheses, sample size, sample characteristics, measurement of exposure, measurement of outcome, perinatal stage at exposure (trimester or infant age), infant age at assessment, confounding variables included, mediating or moderating variables included, main results (unadjusted and adjusted effect size, significance, raw data where required), conclusions.

Risk of bias (quality) assessment

Risk of bias and quality assessment of included studies will be conducted independently by MBD and DP. The Newcastle-Ottawa Scale (NOS) for assessing the quality of non-randomised studies will be utilised as it is specifically designed for use with observational study designs. The NOS assigns a rating of good, fair or poor quality, which will be presented alongside results.

Strategy for data synthesis

A narrative synthesis will be provided, structured around the time of exposure (pre- or post-natal), exploring the key concepts and patterns within the data.
We will also examine the data available for the potential to conduct meta-analysis. Meta-analysis will be conducted if there is sufficient data and sufficient homogeneity in the data. If meta-analysis is not possible, a narrative synthesis will be provided and, where possible, we will calculate individual summary statistics for each study.

Analysis of subgroups or subsets

Studies containing prenatal and postnatal exposure to depressive symptoms will be presented and discussed separately. Outcomes will also be divided into two subgroups: general cognitive development and language development. If sufficient studies are available, we will perform a subgroup analysis of studies which have used the Bayley Scales of Infant Development (BSID) as this is a widely recognised gold-standard measure in HIC settings.

Contact details for further information

Matthew Bluett-Duncan
hlmbluet@liv.ac.uk

Organisational affiliation of the review

University of Liverpool; National Institute of Mental Health and Neurosciences

Review team members and their organisational affiliations

Mr Matthew Bluett-Duncan. Institute of Psychology, Health and Society, University of Liverpool, UK
Dr Thomas Kishore. Department of Clinical Psychology, National Institute of Mental Health and Neurosciences, Bengaluru, India
Ms Divya Patil. Department of Epidemiology, School of Public Health, University of Washington, USA
Professor Helen Sharp. Institute of Psychology, Health and Society, University of Liverpool, UK.

Anticipated or actual start date

09 September 2018

Anticipated completion date

31 March 2019

Funding sources/sponsors

This review is being conducted as part of my Doctoral studies. My dual PhD studentship was awarded by University of Liverpool and NIMHANS, Bengaluru.

Conflicts of interest

Language

English

Country
England, India

Stage of review
Review_Ongoing

Subject index terms status
Subject indexing assigned by CRD

Subject index terms
Asphyxia Neonatorum; Cognition; Depression; Developing Countries; Female; Humans; Income; Infant, Newborn; Pregnancy

Date of registration in PROSPERO
17 December 2018

Date of publication of this version
17 December 2018

Details of any existing review of the same topic by the same authors

Stage of review at time of this submission

**Stage**	**Started**	**Completed**
Preliminary searches	Yes	No
Piloting of the study selection process	No	No
Formal screening of search results against eligibility criteria	No	No
Data extraction	No	No
Risk of bias (quality) assessment	No	No
Data analysis	No	No

Versions
17 December 2018

PROSPERO
This information has been provided by the named contact for this review. CRD has accepted this information in good faith and registered the review in PROSPERO. CRD bears no responsibility or liability for the content of this registration record, any associated files or external websites.
