# Supplementary material for: A systematic review of the association between perinatal depression and cognitive development in infancy in low and middle-income countries
Source: PLoS One. 2021 Jun 25;16(6):e0253790. doi: 10.1371/journal.pone.0253790 (PMC8232443; doi:10.1371/journal.pone.0253790)
Supplement: S1 Table — (DOCX) [file pone.0253790.s003.docx]

**S1 Table.** Antenatal Main Effects

| **Author (Year)** | **Design** | **Cognitive Development** | | | **Language Development** | | |
| --- | --- | --- | --- | --- | --- | --- | --- |
|  |  | **Mean Scores (SD)** | | **Main Effect** | **Mean Scores (SD)** | | **Main Effect** |
|  |  | Depressed | Non-Depressed |  | Depressed | Non-Depressed |  |
| Bandoli et al., (2016) | RCT | **6m:** 90.9 (6.8)  **12m:** 90.8 (10.0) | **6m:** 90.5 (9.2)  **12m:** 93.0 (11.6) | **6m**:  B =-1.96 (4.44-0.52) NS  **12m**:  B = -0.16 (3.54-3.22) NS | N/A | N/A | N/A |
| Breen et al., (2018) | Prospective | **24m:** 83.7 (7.79) | **24m:** 90.0 (6.16) | **24m:**  Effect not given, p <0.001. | **24m:** 83.78 (14.09) | **24m:** 87.12 (9.89) | **24m:** NS |
| Donald et al., (2019) | Prospective | Not Given | Not Given | **24m:**  β = −1.03; (−1.04, −0.12), p = 0.027. | Not given | Not given | Not given |
| Lin et al., (2017) | Prospective | N/A | N/A | N/A | Not given | Not given | **24-30m:**  β = -13.18  (-24.14, -2.22), p = 0.012. |
| Munoz-Rocha et al., (2018) | Prospective | Not given | Not given | **Model 1**  **24-30m:**  β = -2.40 (0.8), p < 0.01  **Model 2:**  **24-30m:**  β = -2.2 (1.1), p = 0.06. | Not given | Not given | **Model 1**  **24-30m:**  β = -2.47 (0.9), p = 0.01  **Model 2**  **24-30m:**  β = -2.17 (1.2), p = 0.08. |
| Murray et al., (2016) | RCT | 81.4 (CI = 78.6, 84.3) | 84.8 (CI = 83.4, 86.2) | **Model 1**  **18m**  F = 4.4, p = 0.04  **Model 2**  **18m**  F = 3.1, p =0.08 | N/A | N/A | N/A |
| Rotheram-Fuller et al., (2018) | RCT | **OS – 36m:**  AND: 1.27 (SE=0.24)  PND: 0.98 (SE=0.18)  A/PND: 1.92 (0.21)  **SS – 36m:**  AND: 6.11 (0.68)  PND: 5.47 (0.52)  A/PND: 5.32 (0.62)  **STS – 36m:**  AND: 5.21 (0.64)  PND: 4.29 (0.49)  A/PND: 4.17 (0.60)  **PPVT – 36m:**  AND: 19.66 (0.79)  PND: 19.20 (0.59)  A/PND: 19.89 (0.73) | **OS – 36m:**  Never: 1.44 (0.15)  **SS – 36m:**  Never: 5.91 (0.45)  **STS – 36m:**  Never: 4.30 (0.42)  **PPVT – 36m:**  Never: 19.79 (0.53) | **OS – 36m:**  All groups NS  **SS – 36m:**  All groups NS.  **STS – 36m:**  All groups NS  **PPVT – 36m:**  All groups NS. | **N/A** | N/A | N/A |
| Tran et al., (2013) | Prospective | **6m:** 97.92 (14.13) | **6m:** 100.03 (12.82) | **6m:** B = -4.80, (95% CI: -9.40, -0.20), p < .05. | N/A | N/A | N/A |

Key: OS = Operation Span; SS = Silly Sounds; STS = Something’s the Same; PND = Postnatal Depression; PPVT = Peabody Picture Vocabulary Test; AND = Antenatal Depression; A/PND = Antenatal and Postnatal Depression.
