# Supplementary material for: A systematic review of the association between perinatal depression and cognitive development in infancy in low and middle-income countries
Source: PLoS One. 2021 Jun 25;16(6):e0253790. doi: 10.1371/journal.pone.0253790 (PMC8232443; doi:10.1371/journal.pone.0253790)
Supplement: S2 Table — (DOCX) [file pone.0253790.s004.docx]

**S2 Table.** Postnatal Main Effects

| **Author (Year)** | **Design** | **Cognitive Development** | | | **Language Development** | | |
| --- | --- | --- | --- | --- | --- | --- | --- |
|  |  | **Mean Scores (SD)** | | **Main Effect** | **Mean Scores (SD)** | | **Main Effect** |
|  |  | Depressed | Non-Depressed |  | Depressed | Non-Depressed |  |
| Ali et al., (2013) | Quasi-Experimental | Not given | Not given | **6m:**  OR= 3.3 (95% CI: 1.1, 9.9)*  **12m:**  OR= 6.8 (95% CI: 3.0, 15.7)* | Not given | Not given | NS (effect not given) |
| Black et al., (2007) | RCT | Not given | Not given | **12m:**  B = .09, *p* > 0.05. | **N/A** | N/A | **N/A** |
| Familiar et al., (2018) | Prospective | Not given | Not given | **12m:**  B = -2.49, (-5.86, 0.88), p = 0.15. | N/A | N/A | N/A |
| Galler et al., (2000) | Prospective | Not given | Not given | **3m:**  F(3, 78 )=2.09; p<0.02.  **6m:**  NS (effect not given) | N/A | N/A | N/A |
| Garman et al., (2019) | RCT-Control Arm | Early: 10.24 (2.72)  Late: 9.59 (3.30)  Chronic: 9.00 (1.84) | Chronic Low: 10.14 (3.03) | **BSID - 18m:**  β = 0.08, p=0.91**  **OS - 36m:**  β =-0.29, p = 0.47**  **SS – 36m:**  β = -0.75, p = 0.54**  **STS – 36m:**  β =-0.11, p = 0.89** | N/A | N/A | N/A |
| Hamadani et al., (2012) | Prospective | 99.7 (10.8) | 100.6 (12.1) | **12m:**  NS (no effect given) | N/A | N/A | N/A |
| Patel et al., (2003) | Prospective | 86.4 (84.1, 88.8) | 90.3 (87.7, 92.9) | **6m:**  OR = 3.3 (1.2, 8.8), p = 0.02.* | N/A | N/A | N/A |
| Quevedo et al., (2012) | Prospective | N/A | N/A | N/A | Postpartum: 107.24 (16.48)  Current: 105.95 (14.08)  Postpartum & Current: 97.43 (15.40) | None: 108.59 (17.00) | **12m:**  B = -2.87 (-5.01; -0.64) p = 0.01*** |
| Tran et al., (2013) | Prospective | 102.88 (14.76) | 98.54 (13.07) | **8w:**  B = 1.26 (-1.02; 3.54), *p* > 0.05.  **6m:**  B = 1.76, (-.09; 3.62), *p* > 0.05. | N/A | N/A | N/A |

* ORs given for likelihood of developmental delay.

** Effect given for early postpartum depressed group only.

*** Effect given for no depression vs any depression. Post-hoc tests revealed effect of postpartum & current group only.

Key: BSID = Bayley Scales of Infant Development; OS = Operation Span; SS = Silly Sounds; STS = Something’s the Same.
