# Supplementary material for: A systematic review of the association between perinatal depression and cognitive development in infancy in low and middle-income countries
Source: PLoS One. 2021 Jun 25;16(6):e0253790. doi: 10.1371/journal.pone.0253790 (PMC8232443; doi:10.1371/journal.pone.0253790)
Supplement: Attachment — Submitted filename: Review Comments_Perinatal Depression and ECD_PONE-D-21-04110.doc [file pone.0253790.s005.doc]

Review of --“ **A systematic review of the association between perinatal depression and cognitive development in infancy in low and middle-income countries**”.

By Matthew Bluett-Duncan, M. Thomas Kishore, Divya M. Patil, Veena A. Satyanarayana and Helen Sharp

The impact of perinatal depression on cognitive outcome of offspring is an important area that still blurred and requires more insights for the future-direction of child-focused interventions. The authors made a good attempt to review the existing assorted literatures on this complex topic and summarize the take-home messages.

The authors very clearly and elaborately described all the steps of the systematic review with references. They also detailed the working definitions of both exposure and outcome variables.

However, they are encouraged to address the following concerns:

•For simplifying the findings, the authors made lots of sub-headings in result and discussion section that caused repetition of similar information This repetition is distorted the flow of reading due to lack of customization. So, some sections could be merged, e.g. “key adjustment sections” does not need to be separately reported. Rather “adjustment information” can be blend with specific studies--- but the tables( 3 & 4) can be presented for better understanding.

• Table 1 & 2 on antenatal & postnatal findings, need to be more informative. At least those cab include positive/negative findings. Otherwise it is difficult to relate results and discussion on antenatal or postnatal findings with several tables in main paper and in supplementary document.

•Sample size of study population and their follow-up percentages need careful checking. For example, sample size and follow up % of the study done by Garman et al in Table-2 is not matching with the journal information. In the journal, at follow-up, 58% children were assessed for developmental measures. Please clarify if the sample size in the tables refers to the numbers of mother-child dyads.

•Reference numbers are wrong in many places e.g. page 17, section 3.3.1, 2^nd^ line, reference 56 will be 58; page 19, ref of Rotheram-Fuller will be 55 etc.

• Throughout the write up please correct “BSID-III”-- It should be **Bayley-III.**

• Finally based on the nature of majority of the studies (mostly prospective or observational and sometimes 2ndary analysis), heterogeneous nature of the exposure/outcome assessments and huge drop-outs at follow up-- it will be better to say “significant association” of antenatal or postnatal depression with cognition, rather than “significant effect”, which is a more robust terminology.
