# Supplementary material for: A systematic review of the association between perinatal depression and cognitive development in infancy in low and middle-income countries
Source: PLoS One. 2021 Jun 25;16(6):e0253790. doi: 10.1371/journal.pone.0253790 (PMC8232443; doi:10.1371/journal.pone.0253790)
Supplement: Attachment — Submitted filename: Response to Reviewers.docx [file pone.0253790.s006.docx]

Matthew Bluett-Duncan

University of Liverpool

Room 2.76, Eleanor Rathbone Building

Liverpool, L69 7ZA, UK

0151 650 5490; [hlmbluet@liverpool.ac.uk](mailto:hlmbluet@liverpool.ac.uk)

21.04.21

To the Editor,

**Re. Revisions to manuscript PONE-D-21-04110**

**A systematic review of the association between perinatal depression and cognitive development in infancy in low and middle-income countries.**

Thank you for considering the revised version of this previously submitted article. I would like to also extend my thanks to both reviewers who offered considered and constructive feedback. Many of the points for revision were valid and have the potential to add value to the article. We have now reviewed the comments fully and made appropriate modifications to the manuscript. Responses to individual comments and the changes made are detailed below.

We would be grateful for your further consideration of the manuscript in the light of the revisions made.

Thank you for your consideration.

Sincerely,

Matthew Bluett-Duncan

PhD Candidate, The University of Liverpool

(Enclosed: Response to reviewers)

**Reviewer 1 comments to the author: no queries raised.**

***Reviewer 2 Comment:* For simplifying the findings, the authors made lots of sub-headings in result and discussion section that caused repetition of similar information This repetition is distorted the flow of reading due to lack of customization. So, some sections could be merged, e.g. “key adjustment sections” does not need to be separately reported. Rather “adjustment information” can be blend with specific studies--- but the tables( 3 & 4) can be presented for better understanding.**

*Author Response:* The “key adjustment” section was originally included as a distinct section as one of the key questions being asked by the review pertains to the ability of each study to isolate the variance in cognitive development that could be attributed to perinatal depression. It was felt that including this as a separate section was therefore justified. However, it is a valid point that its inclusion as a distinct section may not be necessary due to the inclusion of the information elsewhere, and that it serves to break up the flow of the narrative. Following the advice of the reviewer, this section has now been removed but the tables have been retained. The tables are now referenced in the initial descriptions of the antenatal and postnatal studies and the relevance of the information regarding the “key adjustment variables” is referred to and discussed in the discussion section. We believe this retains the emphasis on the importance of these factors but removes some of the repetition.

We also considered whether any of the other sub-sections could be reduced or merged together, particularly in the “Results” section. However, we believe that the way they are currently set is optimal as it allows the reader to clearly distinguish between antenatal (section 3.2) and postnatal studies (section 3.3), and within them, those studies which found significant associations and those that did not. Similarly the layout of the discussion section allows the reader to clearly distinguish between the synthesis of the antenatal studies and the synthesis of the postnatal studies. We also believe that a separate section for methodological considerations is justified due to the emphasis in the review on assessing the robustness of the studies carried out in LMIC setting and the importance of clearly evaluating how different methodological decisions may have impacted results. While it may be possible to merge the discussion regarding methodological issues with the discussion of overall findings, the result would be a loss of clarity for both areas.

***Reviewer 2 Comment:* Table 1 & 2 on antenatal & postnatal findings, need to be more informative. At least those cab include positive/negative findings. Otherwise it is difficult to relate results and discussion on antenatal or postnatal findings with several tables in main paper and in supplementary document.**

*Author Response:* We acknowledge that it would be beneficial to the reader to be able to easily see from the tables whether a study found a significant association or not. Therefore, we have now added a column to tables 1 & 2 to indicate whether or not a significant association has been found. We have not added any further information to these tables as the results section already describes the key findings from each study and we did not want to add in overly repetitive information. The two supplementary tables show a detailed breakdown of the study results for the antenatal and postnatal periods.

***Reviewer 2 Comment:* Sample size of study population and their follow-up percentages need careful checking. For example, sample size and follow up % of the study done by Garman et al in Table-2 is not matching with the journal information. In the journal, at follow-up, 58% children were assessed for developmental measures. Please clarify if the sample size in the tables refers to the numbers of mother-child dyads.**

*Author response:* The reviewer is correct that the follow-up rate for Garman et al was incorrect on the table due to a miscalculation by the lead author. This has now been rectified and all the other follow-up rates checked carefully, with a couple of further minor revisions being made. Follow-up rates were calculated from the information provided by each study regarding the number of mother-infant dyads who completed developmental assessments and/or were included in the final analyses. This has now been clarified in the text as well.

***Reviewer 2 Comments:* Reference numbers are wrong in many places e.g. page 17, section 3.3.1, 2nd line, reference 56 will be 58; page 19, ref of Rotheram-Fuller will be 55 etc.**

*Author Response:* The reviewer was correct in noticing that references were incorrectly labelled in a few instances. References have now been checked and corrected where necessary.

***Reviewer 2 Comments:* Throughout the write up please correct “BSID-III”-- It should be Bayley-III**

*Author Response:* This has now been changed to Bayley-III. BSID-II has also been changed to Bayley II for consistency.

***Reviewer 2 Comments:* Finally based on the nature of majority of the studies (mostly prospective or observational and sometimes 2ndary analysis), heterogeneous nature of the exposure/outcome assessments and huge drop-outs at follow up-- it will be better to say “significant association” of antenatal or postnatal depression with cognition, rather than “significant effect”, which is a more robust terminology.**

*Author Response:* This is a very good point and we have now changed the majority of references to “significant effects” to “significant associations”. There are a few instances where the use of the word “effect” remains more suitable and so it has been retained. Importantly, key sentences that serve to summarise findings in the abstract, discussion and conclusion sections have now been changed to reflect the nature of the studies that were synthesized.
